# mSphere of Influence: Predicting Immune Responses and Susceptibility to Influenza Virus—May the Data Be with You

**DOI:** 10.1128/mSphere.00085-20

**Published:** 2020-03-18

**Authors:** Irene Ramos

**Affiliations:** a Department of Neurology, Icahn School of Medicine at Mount Sinai, New York, New York, USA

**Keywords:** antibodies, data science, influenza vaccines, influenza virus, predictive modeling, transcriptomics

## Abstract

Irene Ramos works in the field of immunology to viral infections. In this mSphere of Influence article, she reflects on how “Global analyses of human immune variation reveal baseline predictors of postvaccination responses” by Tsang et al. (Cell 157:499–513, 2014, https://doi.org/10.1016/j.cell.2014.03.031) and “A crowdsourced analysis to identify ab initio molecular signatures predictive of susceptibility to viral infection” by Fourati et al. (Nat Commun 9:4418, 2018, https://doi.org/10.1038/s41467-018-06735-8) made an impact on her by highlighting the importance of data science methods to understand virus-host interactions.

## Abstract (precis)

Irene Ramos works in the field of immunology to viral infections. In this mSphere of Influence article, she reflects on how “Global analyses of human immune variation reveal baseline predictors of postvaccination responses” by Tsang et al. (Cell 157:499–513, 2014, https://doi.org/10.1016/j.cell.2014.03.031) and “A crowdsourced analysis to identify ab initio molecular signatures predictive of susceptibility to viral infection” by Fourati et al.

## COMMENTARY

The microbiology and immunology fields, as many others, have greatly benefited during the last decade from the rapid generation of biological data that comes with the improved high-throughput technologies. This is linked to an increasing development and accessibility of computational analysis tools. As these factors increase, so do the number of interesting messages hidden in those data and our ability to unravel them. In the last few years, my research interest has been greatly driven by my curiosity about what lies behind those data, and thus, here I chose to feature two papers that have highly influenced my way into this scientific approach and thinking. One paper is titled “Global analyses of human immune variation reveal baseline predictors of postvaccination responses” by Tsang et al. (1). This study was one of the earliest that applied predictive modeling to influenza virus vaccination in humans and found multiple baseline parameters (in addition to others previously known such as age or preexisting antibody titers) that are associated with the ability of the immune system to mount an effective antibody response. A similar motivation drove the second paper I feature in this commentary, but in the context of influenza virus infections: “A crowdsourced analysis to identify ab initio molecular signatures predictive of susceptibility to viral infection” by Fourati et al. (2). In this case, the authors challenged multiple teams of data scientists to develop models to predict susceptibility to infection (as measured by symptoms or virus shedding).

Tsang et al. (1) used a comprehensive data set derived from vaccinated individuals to characterize the immune response to influenza virus vaccination and to develop computational models to predict the magnitude of antibody responses to the vaccine from baseline and early postvaccination time points. Their models also considered the natural variation of the population, which is an important challenge in human biology and immunology. Interestingly, they found some stable prevaccination parameters, mostly based on the frequency of specific cell types (with an important contribution of B cell populations) that could predict the magnitude of the antibody response. Transcriptome data were not predictive of successful vaccination in this study, but a correlation was found between the expression of several genes associated with pattern recognition or interferon signaling before vaccination and the magnitude of the antibody response after vaccination. Therefore, these findings indicate an important role of the innate immune set point in the development of successful humoral immune responses. While data at day 1 of vaccination were not predictive, the authors found an association of early interferon responses with improved antibody responses. A later study by the Human Immunology Project Consortium (HIPC)-Center for Human Immunology (CHI) Signatures Project Team and the HIPC-I Consortium (3) identified transcriptomic signatures predictive of vaccination outcome by increasing the size of the sample population in a multicohort study from multiple geographic locations. Those signatures were mainly associated with B cell antigen receptor (BCR) signaling and inflammatory responses. Other studies have confirmed some of these findings and found additional predictors of increased antibody responses to influenza virus (4–6).

The purpose of the study by Fourati et al. (2) was to identify predictors of susceptibility to infection by influenza virus and other respiratory viruses, using gene expression data from experimental infections in human cohorts, which had been previously screened for negative serology. This study took an extremely interesting approach to interrogate the data: they launched a community challenge by which multiple teams worked on the development of such predictive models. Some of the models were successful at predicting symptoms at days 0 and 1 postinfection, but virus shedding proved more challenging to predict based on the data available. Genes belonging to the heme metabolism pathway, which is known to be associated with inflammation through multiple mechanisms (7), and other genes related to inflammatory responses, were highly represented among the genes associated with more severe symptoms. In addition, genes associated with innate immune activation through interleukin 6 signaling, nuclear factor kappa light-chain enhancer of activated B cells (NF-κB), or components of the complement, as well as interferon gamma-upregulated genes were also associated with the presence of symptoms.

The work reflected in the papers by Tsang et at. (1), Fourati et al. (2), as well as other published studies addressing similar questions have impacted the field, and definitely my way of thinking, in three main ways.

(i) They provided proof of concept that there are baseline traits that are associated with the immunological responses to antigen or susceptibility to virus infection. While more work is necessary to better characterize those signatures, this information will be highly valuable in the future for the identification of biomarkers of immunological fitness and susceptibility toward the ideal implementation of personalized therapeutic and immunization protocols.
(ii) Their findings suggest that events occurring early after vaccination or virus infection determine the ability of the immune system to develop adequate antibody responses or might contribute to disease severity upon infection. Also, they provide insights into the molecular mechanisms associated with outcome. This highlights the need to better understand those early innate immune events that occur following viral infection or vaccination at the cellular and molecular level, which is the main focus of most of my research interest, through the use of *ex vivo* and *in vitro* human tissue or cellular models.
(iii) Experimental biology and data science necessary complement each other as required by the progress of technology. Therefore, these types of studies emphasize the vast amounts of messages hidden in the data and the need to understand and use adequate computational methods to decipher those messages.
